# A 10-Day-Old Murine Model of Coxsackievirus A6 Infection for the Evaluation of Vaccines and Antiviral Drugs

**DOI:** 10.3389/fimmu.2021.665197

**Published:** 2021-05-13

**Authors:** Zaixue Jiang, Yaozhong Zhang, Huayuan Lin, Qingqiu Cheng, Xiaomei Lu, Wenkuan Liu, Rong Zhou, Baimao Zhong, Xingui Tian

**Affiliations:** ^1^ State Key Laboratory of Respiratory Disease, National Clinical Research Center for Respiratory Disease, Guangzhou Institute of Respiratory Disease, The First Affiliated Hospital of Guangzhou Medical University, Guangzhou Medical University, Guangzhou, China; ^2^ Dongguan Institute of Paediatrics, Dongguan Children’s Hospital, The Eighth People’s Hospital of Dongguan City, Guangdong Medical University, Dongguan, China

**Keywords:** HFMD, coxsackievirus A6, murine model, antiviral therapy, vaccine

## Abstract

Coxsackievirus A6 (CVA6) is recognized as a major enterovirus type that can cause severe hand, foot, and mouth disease and spread widely among children. Vaccines and antiviral drugs may be developed more effectively based on a stable and easy-to-operate CVA6 mouse infection model. In this study, a wild CVA6-W strain was sub-cultured in newborn mice of different ages (in days), for adaptation. Therefore, a CVA6-A mouse-adapted strain capable of stably infecting the mice was generated, and a fatal model was built. As the result indicated, CVA6-A could infect the 10-day-old mice to generate higher levels of IFN-γ, IL-6, and IL-10. The mice infected with CVA6-A were treated with IFN-α1b at a higher dose, with complete protection. Based on this strain, an animal model with active immunization was built to evaluate antiviral protection by active immunization. The three-day-old mice were pre-immunized with inactivated CVA6 thereby generating IgM and IgG antibodies within 7 days that enabled complete protection of the pre-immunized mice following the CVA6 virus challenge. There were eight mutations in the genome of CVA6-A than in that of CVA6-W, possibly attributed to the virulence of CVA6 in mice. Briefly, the CVA6 infection model of the 10-day-old mice built herein, may serve as an applicable preclinical evaluation model for CVA6 antiviral drugs and vaccine study.

## Introduction

Hand, foot and mouth disease (HFMD) can jeopardize children’s health in the long term (1). Enterovirus 71 (EV71) was the main pathogen of HFMD, at previous studies (2). As EV71 vaccine has been extensively applied clinically, the infection rate of EV71 has decreased (3), whereas the incidence of HFMD has been elevated over the past few years in some areas (4). The Coxsackie virus A6 (CVA6) becomes a main virus strain that increasingly causes HFMD (5, 6), which has been reported by recent HFMD molecular epidemiology studies; it can even cause severe HFMD, which has been suggested from the relevant clinical case reports (7). CVA6 and EV71 exhibit the similar structures and pertain to the Picornaviridae and enterovirus families (8). Differential diagnoses of clinicians are more difficult to achieve as impacted by the possible similarity between the rash attributed to CVA6 in children and other exudative skin rash diseases, which causes children to developed serious complications (9). So far, there is no specific drug or vaccine to enable treatment or prevention of clinical CVA6 infection. Some antiviral drugs for treating CVA6, such as ribavirin, natural ingredients of herb, IFNα, and some vaccines for preventing CVA6 are under development (10–13). Some candidates showed some effects in preventing and treating CVA6 infection, and suitable animal models are critical to the preclinical studies.

Overall, enterovirus animal models consist of the models of mice, monkeys and transgenic mice (14). SCARB2, a functional receptor of EV71, has an essential effect on EV71 infection, which was indicated from the recent research on enterovirus infection (15). Accordingly, an EV71 transgenic mouse model has been developed, which breaks the age limit of mice infected with enterovirus (16). Thus far, newborn mice infection models of enterovirus CVA6, CVA10, and CVA4 have been studied (17–19), and used to evaluate the effects of antiviral drugs and vaccines. Nevertheless, due to the small age and low weight of newborn mice, the injection dose is limited, and the evaluation effect of antiviral drugs is reduced. Furthermore, the existing infection model of newborn mice is capable of primarily evaluating the immune effect of the HFMD vaccine by using a serum adoptive passive immunization method, and animal models have been rarely studied by simulating active immunity in children (12, 20). Furthermore, an HFMD infection model, capable of receiving the treatment of larger doses of drugs and comprehensively evaluating the early immune effect of children, should be built.

In the present study, the muscle tissues of the younger newborn mice infected with the CVA6 strain were collected to gradually infect the elder newborn mice, and then were exploited to prepare a CVA6-adapted strain capable of stably sacrificing 10-day-old mice. The newborn mouse infection model built here could receive the treatment with larger doses of drugs, which was indicated from the results of *in vitro* injection of recombinant interferon for treatment, so it could be used to assess the preclinical treatment effects of drugs. Moreover, the 3-day-old newborn mice were immunized, and the experiment results (e.g., an *in vitro* challenge) suggested that the built model could be available for evaluating the early immune effect of CVA6. In brief, the CVA6 elder newborn mouse model built can receive the treatment with larger doses of antiviral drugs, and an evaluation model was built for active immunization vaccines. Thus, the present study built an animal model to develop CVA6 therapeutic drugs and vaccines and provide a reference for studies on other types of enterovirus models for elder newborn mice.

## Materials and Methods

### Ethics Statement

The specific pathogen free BALB/c mice were purchased from Guangdong medical laboratory animal center (Guangdong, China). The animal procedures applied here were evaluated and approved by the Laboratory Animal Ethics Committee of Guangdong Medical University (Permit no. GDY2002105). Moreover, the animal experiments were performed by strictly complying with the recommendations of the Guide for the Care and Use of Laboratory Animals of the National Institutes of Health of the United States.

### Viruses and Cell Strains

The CVA6 (Gdula strain) and rhabdomyosarcoma cell line (RD cell) were provided by the American Type Culture Collection (ATCC, USA). In this study, the RD cells cultured in DMEM (Gibco, USA) high-glucose medium supplemented by 10% fetal bovine serum (Gibco, USA) were employed. CVA6 was cultured in RD cells, and the cytopathic effect (CPE) was monitored under the microscope every day for 2 to 4 days. When the cells exhibited typic CPE, the cells and supernatant were collected, frozen, and then thawed three times. Subsequently, they were centrifuged at 12000 rpm for 10 min. Next, the supernatant was re-collected. With the Reed-Muench method (21, 22), the infection titer of CVA6 was detected at 1 × 10^5^ TCID50/0.1 ml, and the CVA6 (Gdula strain) cultured in the cells was termed the CVA6-W strain.

### Screening of CVA6 Mouse-Adapted Strains

To develop a CVA6 adapted strain capable of infecting 10-day-old mice, 100 µl CVA6 of 1 × 10^5^ TCID50/0.1 ml was prepared and diluted by DMEM (high glucose) (Gibco, USA). The three-day-old mice were inoculated with CVA6 of 1 × 10^5^ TCID50 intraperitoneally, and their mental states were observed every day. If hemiplegia occurred in the hind limbs of the mice, the hind limb muscles were sampled, grinded, and then centrifuged at 12000 rpm for 10 min. Next, the supernatant was separated and then filtered *via* a 0.22 µm filter membrane, and intraperitoneally inoculated into the four-day-old mice again. Likewise, the newborn mice at age 6-, 8-, 10-, 12-, and 14-day were inoculated. Their clinical symptoms were scored according to the clinical 5-grade scoring standard as previously reported in a report on CVA16 (23), i.e., 0, 1, 2, 3, and 4, respectively, represent healthy, lethargic or depressed, emaciated and slow-moving, hind limb paralyzed, and dead. Lastly, a CVA6 mouse-adapted strain capable of causing clinical symptoms above grade 3 in the 10-day-old mice was generated and termed as CVA6-A. CVA6-A was diluted in DMEM (high glucose) (Gibco, USA) in a tenfold gradient seven times. Furthermore, the 10-day-old mice (10 per group) were prepared and infected with the diluted CVA6-A; the Reed-Muench method (22, 24) was adopted to detect the LD50 of their CVA6-A at 1 × 10^5^ LD50/0.1 ml. Afterward, to build a negative control, an identical volume of DMEM (high glucose) (Cat. 11995115, Gibco, USA.) was injected into the mice.

### CVA6 Genome Sequencing and Fluorescence Quantitative Reverse Transcription Polymerase Chain Reaction (RT-PCR) Detection

A volume of 100 µl CVA6-W of 1 × 10^5^ TCID50/0.1 ml and 100 µl CVA6-A of 1 × 10^5^ LD50/0.1 ml were prepared. TRIzol (Invitrogen, USA), chloroform, and isopropanol were employed to extract RNA, and the concentration was tested and then quantified to 1 µg. cDNA was synthesized by applying RNA reverse transcription kits (TOYOBO, Japan). The complete length CVA6-A cDNA was amplified by PCR with a primer pair, F1 (5ˊ-TTAAAACAGCCTGTGGGTTGCA-3ˊ) and R1 (5ˊ-GCTATTCTGGTTATAACAAATTTAC-3ˊ) with KOD FX enzyme (TOYOBO, Japan). The PCR product was purified and then cloned into pcDNA3.1(+) with In-fusion Cloning Kit (TaKaRa, China). Thereafter, a number of positive plasmid clones were screened and then sequenced. The CVA6-A sequences were aligned with CVA6-W genome, and the mutations were identified.

CVA6 fluorescent quantitative RT-PCR (qRT-PCR) detection primers (e.g., 5ˊ-CGATGAGAACCTGATTGA-3ˊ and 5ˊ-CCTCCACAACTCCTACTA-3ˊ) and β-actin internal reference detection primers (e.g., 5ˊ-CGTTGACATCCGTAAAGACC-3ˊ and 5ˊ-AACAGTCCGCCTAGAAGCAC-3), were employed to quantitatively detect the RNA content in CVA6-W and CVA6-A, respectively. Specifically, the reaction conditions of qRT-PCR comprised 95°C pre-denaturation for 2 min, 95°C denaturation for 15 s, and 60°C annealing for 1 min, in 40 cycles. The standard curve equation was written as y = −3.29467X+34.46449, R2 = 0.99501, and the detection limit reached 5.597 copies/µl. The final detection of the viral load of CVA6-W in the cells was log10 copies/ml=8.3266, and that of CVA6-A in the muscle tissues of the mice was log10 copies/ml=7.9493.

### Homology Modeling

The primary amino acid sequences of CVA6-W and CVA6-A capsid proteins (VP2, VP3, VP1) were submitted to the SWISS-MODEL workspace for homology modeling. The highest-quality templates were selected for model building. The models were built based on the target-template alignment using ProMod3. Coordinates that were conserved between the target and the template were copied from the template to the model.

### Establishment of the Infection Model of 10-Day-Old Mice

To perform a challenge experiment, the mice were divided into three groups. When the first group was being tested, 15 10-day-old mice were divided equally into three groups. Based on the intraperitoneal injection, the first group was injected with 100 µl CVA6-W of 1 × 10^5^ TCID50/0.1 ml; the second was injected with 100 µl CVA6-A of 1 × 10^5^ LD50/0.1 ml; the third received 100 µl of DMEM (high glucose) and was set as the negative control. The states of the mice were observed daily, and those with clinical scores over 4 in the CVA6-A and other groups, 10 days after the challenge experiment, were sacrificed humanely. The tissues of the skeletal muscles, cerebellums, hearts, livers, spleens, lungs, kidneys, and small intestines of the mice were split into two parts. One part was soaked in 4% paraformaldehyde, and the other was stored in a refrigerator at −80°C. In addition, fluorescence quantitative PCR (qPCR) was performed to detect the CVA6 viral loads in the tissues. When the challenge experiment was performed in the second group, 20 10-day-old mice were intraperitoneally injected with 100 µl 1 × 10^5^ LD50/0.1 ml CVA6-A. The blood, leg muscles, cerebellums, hearts, spleens, lungs, and small intestines of the five mice sacrificed humanely every day were taken and then stored in a refrigerator at −80°C. Next, fluorescence qPCR was performed to detect the CVA6 viral loads in the tissues. The challenge experiment in the third group was identical to that performed on the first group, in which the blood samples of all mice only 4 days after the challenge experiment, were collected to separate the serum.

### Cytokines Detection

This study employed Cytokine ELISA detection kits (Shanghai Enzyme Link Biotechnology Co., Ltd., China) to detect the concentrations of IL-4, IL-6, IL-10, and IFN-γ in the serum prepared by performing a challenge experiment on the third group of mice. Following the kit instructions, the serum was added to the ELISA plate to be tested. Each serum sample was tested three times. Horseradish peroxidase-labeled streptavidin and TMB were adopted to enable color development. In addition, a microplate reader with an optical density value of 450 nm was adopted to detect the absorbance, and a standard curve was plotted to calculate the cytokine concentration of each serum sample. The detection sensitivity of this kit for IL-4, IL-10, and IFN-γ reached 1.0 pg/ml, while that for IL-6 reached 0.1 pg/ml.

### Pathological Section and Immunohistochemical Detection

The tissues prepared by performing the challenge experiment on the first group were embedded in paraffin after being dehydrated and permeabilized and then cut into 4-µm sections. Next, the sections were stained with hematoxylin and eosin for observation. Moreover, a new paraffin section was prepared and soaked in citrate buffer solution. It was heated to 90°C in a microwave and then kept warm for 20 min. To complete the dewaxing and the dehydration, the sections were soaked in xylene I, xylene II, absolute ethanol, 95% ethanol, 90% ethanol, as well as 80% ethanol. By using 3% H_2_O_2_, the endogenous peroxidase activity of the tissues in the sections decreased. After being washed with distilled water three times, the sections were soaked in PBS solution, heated to 90°C in a microwave and then kept warm for 10 min. The serum was removed after the solution was sealed with goat serum for 15 min at ambient temperature. The CVA6 VP2 rabbit polyclonal antibody (Gene Tex, USA) was diluted with PBS solution at 1:100 and then the tissues were dropped onto the sections. Subsequently, the sections were incubated in a wet box at 4°C overnight. After the sections were washed three times with PBS solution on the next day, the diluted HRP-goat anti-rabbit-IgG secondary antibodies (CWBIO, China) were added, and then the sections were incubated for 2 h at ambient temperature. After being washed with PBS six times, they were added with diluted DAB solution for color development, washed with distilled water, and then counterstained with hematoxylin to observe the results under the microscope.

### IFN-α1b Anti-CVA6 Infection *In Vitro*

RD cells were cultured in a 96-well cell culture plate, and the cell culture solution was removed under the cell production confluence of 70%. To the respective cell well was added 100 µl CVA6-W of 100TCID50/0.1 ml and then incubated in 5% CO_2_ for 1 h at 37°C and DMEM maintenance medium (2% FBS) was adopted to dilute IFN-α1b, so the concentration of IFN-α1b was obtained as 1.875, 0.9375, 0.4687 µg/ml, 0.2343 µg/ml, and 0.1172 µg/ml, respectively, for dilutions 1 to 5. After the virus solution was removed, IFN-α1b of different concentrations after three times testing, was added to the cell wells to observe the cell status at 24, 48, 72, and 96 h. After 96 h of IFN-α1b anti-CVA6-W infection, CCK8 kits (Kangwei Shiji, China) were employed to detect the survival rate of the cells. Furthermore, in these experiments, RD cells without virus inoculation were grouped as negative controls.

### IFN-α1b Anti-CVA6 Infection *In Vivo*

Fifteen 10-day-old mice were divided into three groups. Each mouse in the first group was intraperitoneally injected with 100 μl CVA6-A of 1 × 10^5^ LD50/0.1 ml, as well as with 200 μl IFN-α1b (30 µg/ml) after being infected. The mental states of mice were observed daily and then they were sacrificed humanely on the sixth day after being infected. The inoculation method and the dose of the second group were identical to those of the first group. In addition, the mice were intraperitoneally injected with 200 µl PBS at 24 h and 48 h after the virus infection, and then sacrificed humanely when they developed grade three clinical symptoms. The respective mouse in the third group was intraperitoneally injected with 100 µl PBS and 200 µl PBS at 24 h and 48 h after the first injection, and then sacrificed humanely after being observed for 6 days. The tissues of the skeletal muscles, hearts, livers, spleens, lungs, kidneys, brains, and the small intestines of the mice of each group were collected and then stored in a refrigerator at −80°C. Furthermore, fluorescence qPCR was employed to detect the CVA6 viral loads in the tissues.

### Inactivated Virus Vaccination and Challenge

Considerable CVA6-W cultured RD cells were purified, viral loads were detected, and then inactivated at 56°C for 30 min. Twenty-three-day-old mice were divided into four equal groups. Each mouse in the first and third groups was intraperitoneally inoculated with 10 µl PBS, and each mouse in the second and fourth groups was inoculated using the identical method and then injected with an identical volume of inactivated CVA6-W. The states of the mice were observed daily until they were older than 10 days, and then an *in vivo* challenge experiment was performed. The respective mouse in the third and fourth groups was injected intraperitoneally with 100 µl CVA6-A of 1 × 10^5^ LD50/0.1 ml, and each mouse in the first and second groups was injected intraperitoneally with 100 µl PBS. The statuses of the mice were observed daily, and all mice were sacrificed humanely. The tissues of the cerebellums, myocardium, lungs, leg muscles, small intestines, and blood samples of the mice were taken and then stored in a refrigerator at −80°C. Furthermore, fluorescence qPCR was adopted to detect the CVA6 viral loads in the tissues.

Fifteen 3-day-old mice were divided into three equal groups. The first and second groups were injected subcutaneously with inactivated CVA6-W and CVA6-A with identical viral loads *via* their backs. The third group was injected with an identical volume of PBS as the negative control. The serum was collected from the mice 7 and 14 days later. The collected serum was stored at −20°C for indirect ELISA and *in vitro* neutralization test.

### Indirect ELISA

Purified CVA6-W of 1×104 TCID50 were diluted with the ELISA coating buffer (pH 9.6) (Solarbio, China) and added into each well of 96-well ELISA plate (Costar, USA). All plates were stored at 4°C temperature overnight and then washed with PBST (pH 7.2–7.4) (Solarbio, China). Then, 100 µl blocking buffer (5% bovine serum albumin [BSA] in PBST) (Solarbio, China) were added into each well and stored at 37°C for 1 h. After the plate was washed, the 10-fold serially diluted antisera in blocking buffer were added into the plate and incubated at 37°C for 1 h. The plates were washed three times with PBST and incubated with a 1:8,000 dilution of horseradish peroxidase (HRP)-conjugated goat anti-mouse IgG(H+L) (CWBIO, China) secondary antibody or HRP-conjugated goat anti-mouse IgM (Abcam, UK) at 37°C for 1 h. After the plate was washed four times, 100 µl TMB Single-Component Substrate solution (Solarbio, China) were added into each well of the plates for 10 min. Then 100 µl ELISA Stop Solution (Solarbio, China) were added into each well and the results were analyzed with an ELISA plate reader (Multiskan MK3; Thermo Scientific) at 450 nm.

### *In Vitro* Neutralization Test

RD cell suspension with a cell density of 1 × 10^5^ cells/ml was prepared. One hundred microliter of the cell suspension was added into the respective well of a 96-well cell culture plate, and then cultured at 37°C and 5% CO_2_ till the cell growth confluence reached 70% or more. Twenty-five microliter serum of immune-inactivated CVA6-W, CVA6-A, and PBS were added into the respective well of the first column of a new 96-well cell culture plate in turn. To be specific, each serum was tested three times. Moreover, 50 µl DMEM supplemented by 2% FBS was added to each cell well in columns of 2 to 12. Seventy-five microliter medium with an identical serum concentration was added to the cell wells in the first row with an 8-well pipette and then mixed effectively. Subsequently, 50 µl of the mixed solution was drawn to dilute the medium from columns 2 to 11 at 1:2. Fifty microliter CVA6-W of 200TCID50/0.1 ml was added to all cell wells, and the cell culture plate was incubated at 37°C and 5% CO_2_ for 1 h. The culture solution in the RD cell culture plate was removed, and the incubated serum-virus mixture was transferred to the RD cell culture plate and then cultured at 37°C and 5% CO_2_ for 7 days. The statuses of the cells were observed daily, so the serum concentration at which 50% of the cells did not exert any CPE effect was the highest serum titer.

### Statistical Analysis

GraphPad Prism 8 software was employed to statistically analyze the survival rate of the newborn mice, the neutralizing antibody titer, the vaccine immune antibody titer, as well as the viral load. Furthermore, analysis and variance analysis were conducted on all the acquired data by performing the t test, and P <0.05 indicated statistical significance.

## Results

### Establishment of a CVA6 Infection Model of the Older Mice

**Figure 1:**
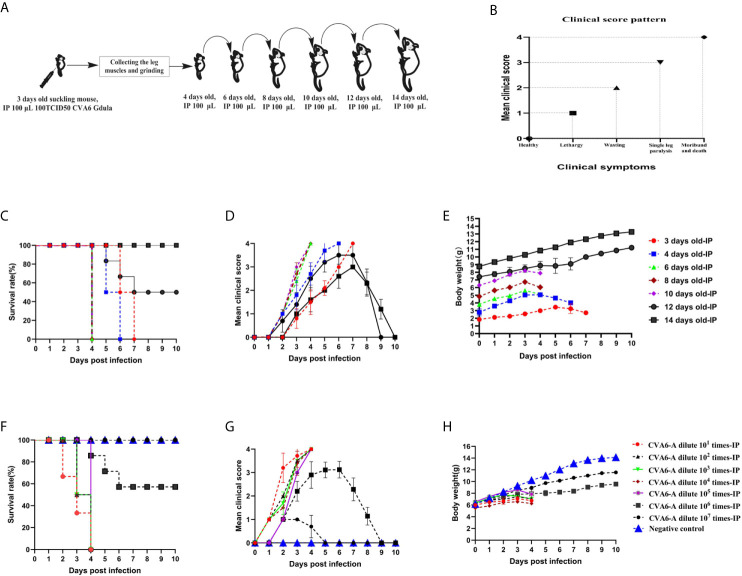
Obtaining the coxsackievirus A6 (CVA6) adapted strain and establishment of the older suckling mice model. The adaptation diagram of CVA6 infecting suckling mice of different ages **(A)** according to the clinical symptom score standard **(B)**. For each group of mice (n = 10), the survival rates **(C)**, clinical scores **(D)**, and body weights **(E)** are the main monitoring indicators in the CVA6 infected suckling mice of different ages, *via* intraperitoneal injection. The LD50 of CVA6 adapted strain for 10-day-old suckling mice (10 per group, 7 groups) that were intraperitoneally injected with seven concentrations of virus fluid was calculated to be nearly 105.0 TCID50/mouse. The survival rates **(F)**, clinical scores **(G)**, and body weights **(H)** were identical to the above.

To develop a CVA6 adapted strain capable of infecting older newborn mice, CVA6-W was used to infect three-day-old mice first, the leg muscles of the mice with typical clinical symptoms were collected and grinded to infect 6-, 8-, 10-, 12-, and 14-day-old mice (**Figure 1A**). The pattern diagram shows the clinical scores (**Figure 1B**). As indicated in **Figure 1C**, 3-day-old mice infected with CVA6-W died 7 days post-infection, with typical hindlimb paralysis and respiratory failure (**Figure 1D**), as well as loss of weight after 5 days post-infection (**Figure 1E**). With the continuous adaptation of CVA6-W in mice of different ages (in days), a CVA6 mouse adapted strain (CVA6-A), able to stably infect 10-day-old suckling mice was developed. This mouse-adapted strain was lethal to some of the 12-day-old mice, instead of the 14-day-old mice, as illustrated in **Figures 1C–E**. As shown in **Figure 1D**, CVA6-A injection caused mild symptoms in 14-day-old mice, with two to three clinical scores, 7 days post-infection.

To determine the LD50 of CVA6-A that infected the 10-day-old mice, the generated CVA6-A was diluted in a tenfold gradient; 100 µl virus solution of each dilution gradient was intraperitoneally inoculated into the 10-day-old mice, and their states were observed. The survival curve, clinical performance score, and body weight of the mice infected with the virus solution of the respective gradient are presented in **Figures 1F–H**, respectively. All (100%) the mice injected with 100 µl CVA6-A diluted 105-fold had the highest clinical score of 4 and died 4 days post-infection (**Figures 1F, G**) with decreasing body weight on day 4 (**Figure 1H**).

**Figure 2:**
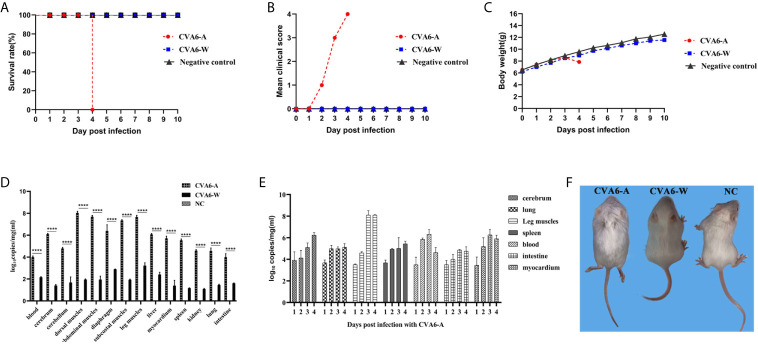
The analysis of CVA6 infection features in 10-day-old suckling mice. When calculating the CVA6 load titer, qPCR was performed to measure the identical volume of CVA6-W (Gdula) and CVA6-A (adapted strain). Similar viral loads were obtained for both CVA6-W and CVA6-A that were intraperitoneally injected to infect 10-day-old suckling mice (5 per group). The survival rates **(A)**, clinical scores **(B)**, and body weight changes **(C)** were measured at the post-infected state. To detect the viral load *via* qPCR **(D)**, blood, brain, skeletal muscles, heart, liver, lung, and other tissues were collected from the mice. The cerebrum, lung, leg muscle, spleen, blood, intestine, and myocardium tissues of the mice, on various days (n = 5), were injected with CVA6-A and were measured using an identical method by CVA6 qRT-PCR **(E)**. Similar viral loads were obtained for CVA6-A, which caused the 10-day-old suckling mice to develop hind limb paralysis, unlike CVA6-W **(F)**. NC served as the negative control group of mice injected with PBS. ****p < 0.0001.

Then we established the 10-day-old mice model of CVA6-A infection. We monitored the weight change and the survival curve daily after injection with 100 µl CVA6-A of 1 × 10^5^ LD50/0.1 ml. The mortality rate of the mice injected with 100 µl CVA6-A of 1 × 10^5^ LD50/0.1 ml was 100%, 4 days post-infection (**Figures 2A, B** **)**, and their body weight was significantly reduced (**Figure 2C**). In contrast, the 10-day-old mice injected with CVA6-W or DMEM injection (negative control group) had no symptom during the entire study period, 10 days post-infection (**Figures 2A–C** **)**.

As indicated in the result of the fluorescence qPCR detection on the newborn mice tissues of the CVA6-A, CVA6-W, and the negative control groups, the skeletal muscles of the group infected with CVA6-A achieved the highest viral load. For the identical tissues, the viral loads of the CVA6-W group were significantly lower than those of the CVA6-A group (**Figure 2D**). To more specifically determine the biological characteristics of the mice infected with CVA6-A, fluorescence qRT-PCR detection was performed on the tissues of the newborn mice infected at different ages (in days). As revealed by the results, the viral load in their blood was the highest on the second day of CVA6-A infection; the viral load in the leg muscles increased sharply and exceeded that in the other tissues on the third day; the CVA6 viral loads in the blood, small intestines, and myocardium began to fall on the fourth day (**Figure 2E**). All (100%) of the mice intraperitoneally injected with the 100 µl CVA6-A diluted 105-fold developed clinical symptoms of hind limb paralysis on day 4 post-infection, whereas the CVA6-W group that was injected with the same viral load had no clinical symptom, as shown in **Figure 2F**.

**Figure 3:**
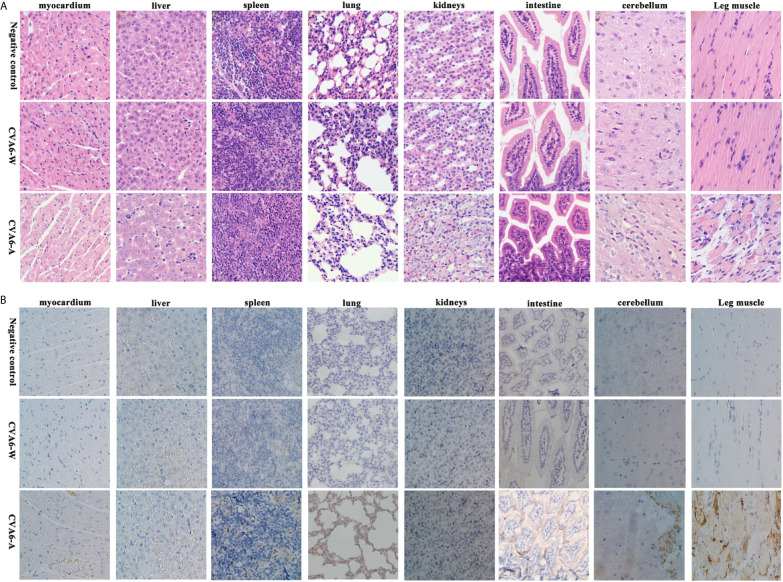
The analysis of the histopathological and immunohistochemical (IHC) examination (200×). Ten-day-old suckling mice were intraperitoneally injected with a lethal dose of coxsackievirus A6 (CVA6)-A, a similar viral load of CVA6-W, as well as an identical volume of DMEM (NC group). The myocardium, liver, spleen, lung, kidneys, intestine, cerebrum, and leg muscle tissues were made into pathological sections and observed **(A)**. IHC examination was performed using anti-CVA6 VP2 antibody **(B)**.

As revealed by the results of the pathological sections and immunohistochemical examinations of the tissues of the myocardium, liver, spleen, lung, kidneys, intestine, cerebrum, and leg muscles (**Figure 3A**), CVA6-A could cause muscle fiber breakage in older newborn mice. Furthermore, typical pathological variations were not identified in the tissues of the CVA6-W group and the negative controls. As demonstrated by the immunohistochemical results, specific stain could be observed in the lungs, cerebellums, and leg muscles of the suckling mice that were infected with CVA6-A, instead of in the other tissues or the tissues of the other groups (**Figure 3B**).

**Figure 4:**
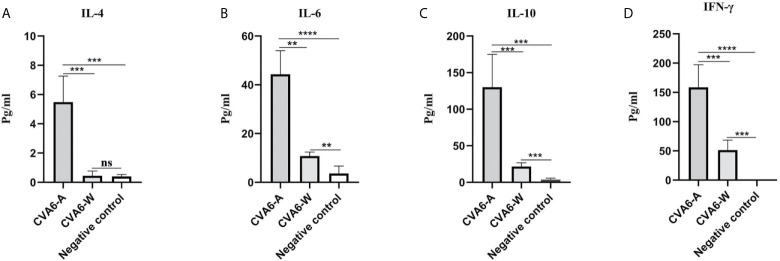
Cytokine expression in the coxsackievirus A6 (CVA6)-A and CVA6-W infected mice. Here, 10-day-old suckling mice were inoculated with a similar load of CVA6-A and CVA6-W. The expression levels of IL-4 **(A)**, IL-6 **(B)**, IL-10 **(C)**, and IFN-γ **(D)** were detected by using enzyme-linked immunosorbent assay from the serum of the post-infected CVA6-A and CVA6-W 4 d.p.i. (n = 3). **p < 0.01, ***P < 0.001, ****P < 0.0001, n.s., insignificant difference.

To determine the cytokine levels in the mice infected with CVA6-A, the concentrations of IL-4, IL-6, IL-10, and IFN-γ in the mouse serum of the CVA6-A group, CVA6-W group, and the negative control group were detected. The concentrations of IL-4, IL-6, IL-10, and IFN-γ in the serum of the CVA6-A group were significantly higher than those in the other two groups. The IL-4 concentrations in the CVA6-A group were 10-fold higher than those in the CVA6-W or the negative control group (**Figure 4A**). The IL-6, IL-10, and IFN-γ concentrations in the CVA6-A group were 5-fold, 6-fold, and 3-fold higher than those in the CVA6-W group, respectively; 12-fold, 60-fold, and over 200-fold higher than those in the control group (**Figures 4B–D**). It was interesting to find that CVA6-W infection induced significantly increasing IL-6 (**Figure 4B**), IL-10 (**Figure 4C**), and IFN-γ (**Figure 4D**), but not IL-4 (**Figure 4A**) in the sera, compared with those of the negative control group.

### Drug Evaluation Model of IFN-α1b Anti-CVA6 Infection

**Figure 5:**
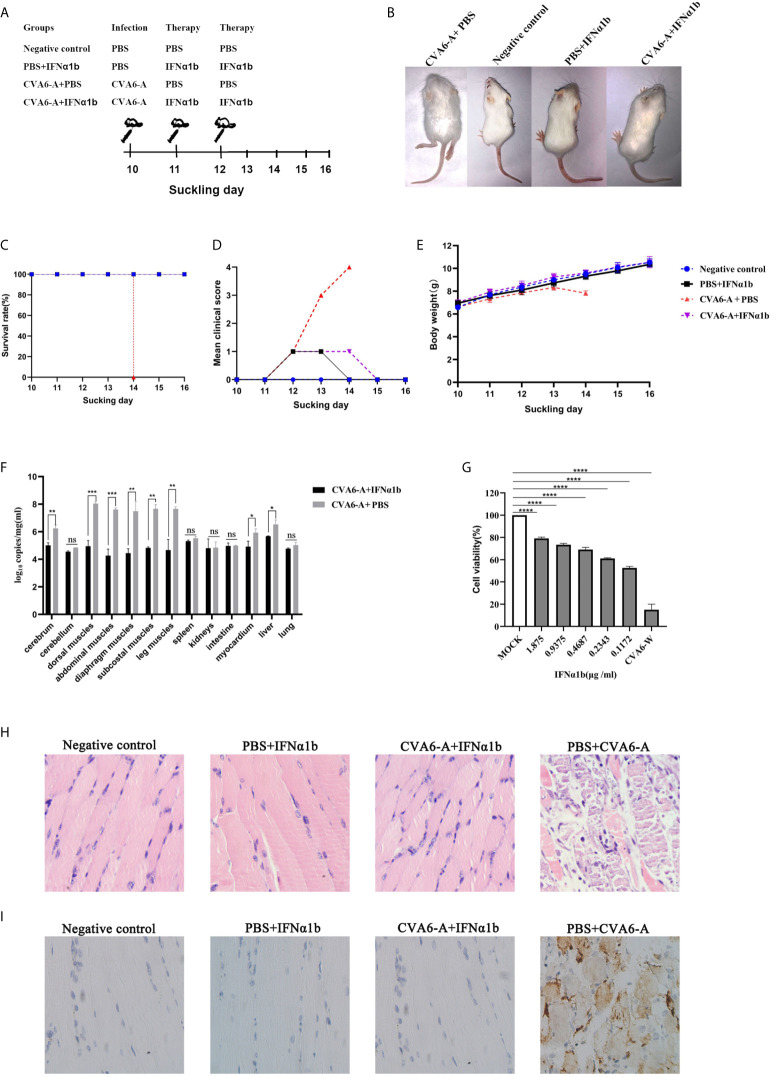
The therapy effects of IFN-α1b against coxsackievirus A6 (CVA6) infection in 10-day-old suckling mice. **(A)** The diagram showing IFN-α1b treatment of CVA6-A infection among 10-day-old suckling mice. The infected mice (n = 5) were given IFN-α1b therapy twice after CVA6-A infection, and the infection control group mice (n = 5) were injected with an identical volume of PBS instead of IFN-α1b after CVA6-A infection. The negative control group and IFN-α1b control group mice were not infected with CVA6-A. After being treated twice with IFN-α1b, no differences were observed between the suckling mice injected with CVA6-A and IFN-α1b and the negative controls **(B)**. The survival rates **(C)**, clinical scores **(D)**, and body weight change **(E)** were the main factors measured daily until 6 dpi. The main tissues of the infected and treatment group mice were collected to detect the viral loads by using qPCR, and posttreatment with IFN-α1b, in which the viral load was identified to be significantly lower than that of the untreated group **(F)**. In vitro, after CVA6-W infected RD cells, IFN-α1b was suggested to inhibit CVA6-W activities *via* CCK-8 detection **(G)**. *p < 0.05; **p < 0.01; ***p < 0.001, ****p < 0.0001. n.s., insignificant result. Ten-day-old suckling mice were intraperitoneally injected with a lethal dose of CVA6-A, a similar viral load of CVA6-W, as well as an identical volume of DMEM. The histopathological **(H)** and immunohistochemical (IHC) **(I)** examinations of the leg muscle tissues of the CVA6-A infected mice treated with IFN-α1b or PBS (200×). The leg muscles were made into pathological sections and then observed **(H)**. IHC examination was performed using anti-CVA6 VP2 antibody **(I)**.

In vivo, 100 µl CVA6 adapted strain of 1 × 10^5^ LD50/0.1 ml or 100 µl PBS, as the control, was injected intraperitoneally into 10-day-old mice, and the mice were subcutaneously injected with 200 µl IFN-α1b (or PBS as the control) 24 h and 48 h post-infection as treatment (**Figure 5A**). The mice were raised till they were 16-day-old and observed for their states daily. After being treated twice with IFN-α1b, on day 4 post-infection, the suckling mice injected with CVA6-A survived and had no symptom; the mice in two control groups without CVA6-A infection also survived and had no symptom; in contrast, the mice of CVA6-A+PBS group (injected with CVA6-A and treated with PBS) showed two leg paralysis and were moribund on day 4 post-infection (**Figure 5B**).

All (100%) of the mice in the CVA6-A+IFN-α1b group (therapy group) survived 6 days post-infection (**Figure 5C**). The survival curves of all newborn mice are presented in **Figure 5C**. The mice that were injected with CVA6-A and PBS displayed grade 1 (lethargy); grade 3 (single leg paralysis); and grade 4 (moribund and death) clinical symptoms on days 2, 3, and 4 post-infection, respectively (**Figure 5D**). In contrast, the mice in the therapy group displayed grade 1 clinical symptom for two days after 48 h infection and recovered to grade 0 (health) finally. The mice of PBS+IFN-α1b group displayed grade 1 clinical symptom for one day after the two times treatment and recovered to being healthy. The mice in the negative control group did not show any clinical symptom (**Figure 5D**). As demonstrated by the weight proliferation curve, the weight of the IFN-α1b-treated mice increased, and the mice in the untreated group lost their weight day 4 post-infection, with the CVA6 adapted strain (**Figure 5E**). The CVA6 genome copies in the tissues of the cerebrum, cerebellum, dorsal muscles, abdominal muscles, diaphragm muscles, subcostal muscles, leg muscles, spleen, kidneys, intestine, myocardium, liver, and lung were quantified by qRT-PCR (**Figure 5F**). The result showed that IFN-α1b treatment significantly reduced 10- to 1000-fold the CVA6 copies, *in vivo*, especially in the tissues of cerebrum, dorsal muscles, abdominal muscles, diaphragm muscles, subcostal muscles, the leg muscles, myocardium, and liver (**Figure 5F**). To determine the anti-CVA6 infection effect of IFN-α1b, RD cells were cultured *in vitro*, inoculated with CVA6-W of 100 TCID50, inoculated with IFN-α1b, and then cultured for 7 days. The CCK8 detection kit was adopted to detect the survival rate of the cells, and the results indicated that the IFN-α1b of 0.1172 µg/ml can inhibit the infection of RD cells by CVA6 *in vitro* (**Figure 5G**).

According to the results in **Figure 5F**, we selected the leg muscles of mice for pathological section and immunohistochemical detection. The muscle fibers of the mice in the negative control group and PBS+IFN-α1b group were intact (**Figure 5H**). The muscle fibers of the IFN-α1b therapy group (CVA6-A+IFN-α1b) were also intact, whereas that of the CVA6-A+PBS group were breaking (**Figure 5H**). CVA6 antigen were detected in the mouse muscles of CVA6-A+PBS group, but not in those of IFN-α1b therapy, negative control, and PBS+IFN-α1b groups (**Figure 5I**).

Given the stated results, IFN-α1b significantly impacted anti-CVA6 infection. Compared with the existing models for newborn mice infected with CVA6, this model, built to include the older newborn mice infected with CVA6, could receive the higher doses of drugs, and the weight and mental state of the mice injected with higher doses of IFN-α1b were significantly different from those in the control group.

### Evaluation Model of Anti-CVA6 Infection Vaccine

**Figure 6:**
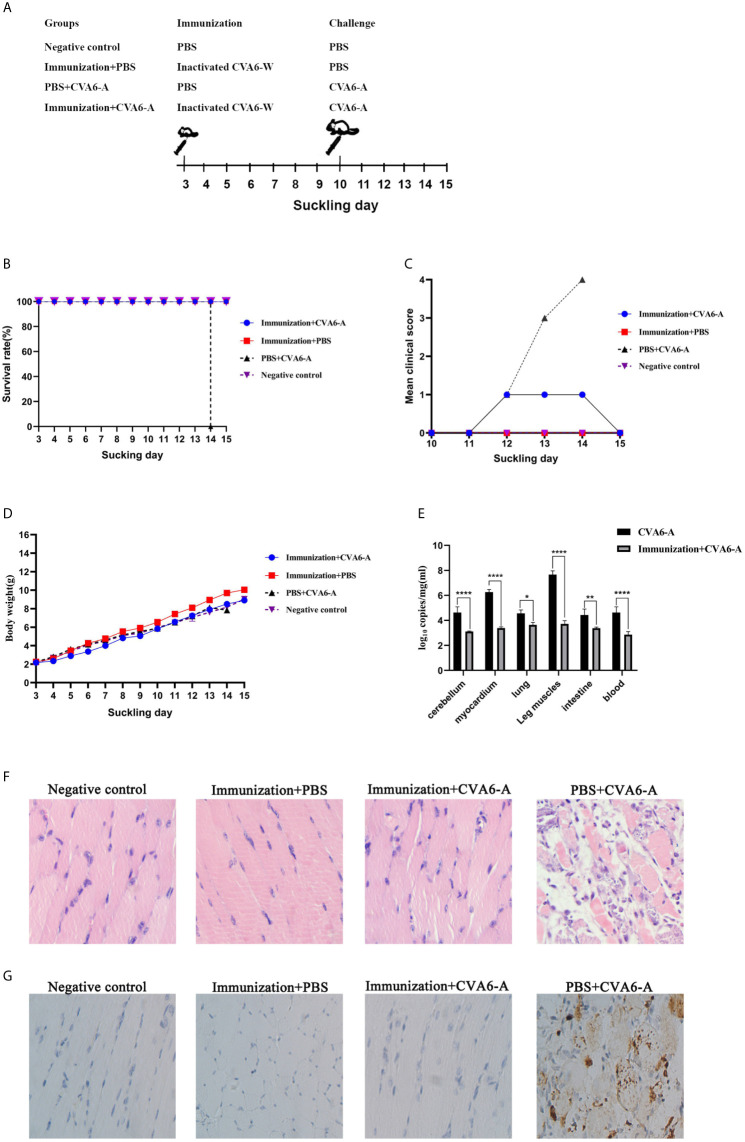
Immunization and challenge of young suckling mice. **(A)** The diagram of the 10-day-old suckling mice challenged with coxsackievirus A6 (CVA6)-A pre-immunization, using inactivated CVA6-W. Two out of four groups of three-day-old mice (5 per group) were immunized with inactivated CVA6-W, while the other two groups were inoculated with PBS. As above, the survival rates, body weights, and the clinical scores were detected daily **(B–D)** after the challenge. The viral load in mice cerebellum, myocardium, lung, leg muscles, intestine, and the blood samples of the mice were detected by using qPCR **(E)**. **(F)** The histopathological and **(G)** immunohistochemical examinations of the tissues of the leg muscle of the challenged mice that were pre-immunized with CV6-A or PBS. All slice results were observed under 200×. *p < 0.05, **p < 0.01, ****p < 0.0001.

To test the mental state and assess the weight gain of the mice vaccinated with CVA6-W inactivated virus, three-day-old newborn mice were selected as the targets of vaccination, as an attempt to simulate the immune response induced by immunization of children with CVA6 vaccine. In addition, the results demonstrated that CVA6-W inactivated virus did not affect the weight gain of the inoculated mice. On the seventh day after the immunization, the mice were intraperitoneally injected with 100 µl CVA6-A of 1 × 10^5^ LD50/0.1 ml (**Figure 6A**), and their mental states and weight changes were observed. Ninety-six hours after the challenge experiment, the group of mice inoculated with CVA6-W inactivated virus only developed depression but recovered on the fifth day, whereas the group of mice immunized with CVA6-W inactivated virus but without CVA6-A challenge showed similar clinical manifestation to that of the negative control. The group of mice immunized with PBS achieved typical symptoms (e.g., hindlimb paralysis and respiratory failure) 96 h after being infected with the identical batch of CVA6-A (**Figures 6B–D**). According to the further detection of the CVA6 loads of the tissues of the myocardium, leg muscles, and cerebellums of the mice in the challenged groups, respectively immunized with CVA6-W inactivated virus and PBS, the content of CVA6 in the leg muscles and cerebellums of the mice immunized with CVA6-W inactivated virus was significantly lower than those immunized with PBS (**Figure 6E**).

The results of the pathological section of the muscle fiber of the mice that were immunized were similar to those of the negative group mice, the muscle fibers were not broken. Whereas the muscle fiber of the mice injected with PBS and CVA6-A was broken (**Figure 6F**). The results of immunohistochemistry showed that CVA6 was only detected in the muscles of the mice that were injected with PBS, compared with those of the other group (**Figure 6G**).

These results demonstrate that the newborn mice immunized with CVA6-W inactivated virus could neutralize CVA6-A infection.

**Figure 7:**
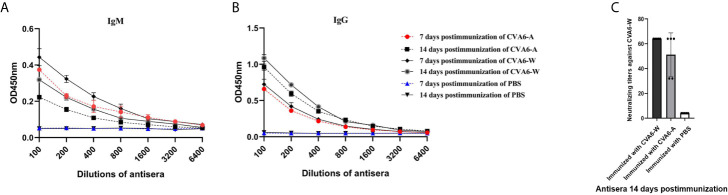
Antibody responses of the 3-day-old mice immunized with inactivated coxsackievirus A6 (CVA6)-W, CVA6-A, or PBS. Three groups of three-day-old mice (5 per group) were immunized with inactivated CVA6-W, inactivated CVA6-A, or PBS. Their sera were collected 7 and 14 days later, respectively. The levels of IgM **(A)** and IgG **(B)** were detected by the enzyme-linked immunosorbent assay method. **(C)** All the sera collected 14 days post-immunization were used to detect the neutralization titer against CVA6-W by performing *in vitro* micro-neutralization test. The inactivated CVA6-A or CVA6-W induced the suckling mice to produce neutralizing antibodies against CVA6-W.

To check whether the antigenicity of CVA6-A changed or not compared to CVA6-W, CVA6-W and CVA6-A inactivated viruses with the identical viral load were adopted to immunize 3-day-old mice. As indicated from the detected changes of IgM and IgG in the mice 7-day and 14-day after the immunization, the IgM antibody titers of the groups immunized with CVA6-W and CVA6-A began to decrease 14-day post-immunization compared to that on day 7 post-immunization; whereas IgG antibody titer changes displayed the opposed trend (**Figures 7A, B** **)**. It was also important to identify that CVA6-specific IgG could be detected on day 7 post-immunization. In vitro micro-neutralization test was done using antisera to neutralize CVA6-W infection in RD cells. Inactivated CVA6-W induced neutralizing antibodies on day 14 post-immunization. Importantly, CVA6-A immunization of 3-day-old mice also induced similar titers of neutralizing antibodies against CVA6-W on day 14 post-immunization (**Figure 7C**). Due to the failure of CVA6-A in triggering RD cells to produce CPE, the cellular micro-neutralization test using antisera to neutralize CVA6-A infection in RD cells was not conducted.

### Comparative Genomic Analysis of CVA6-A With CVA6-W Strains

**Figure 8:**
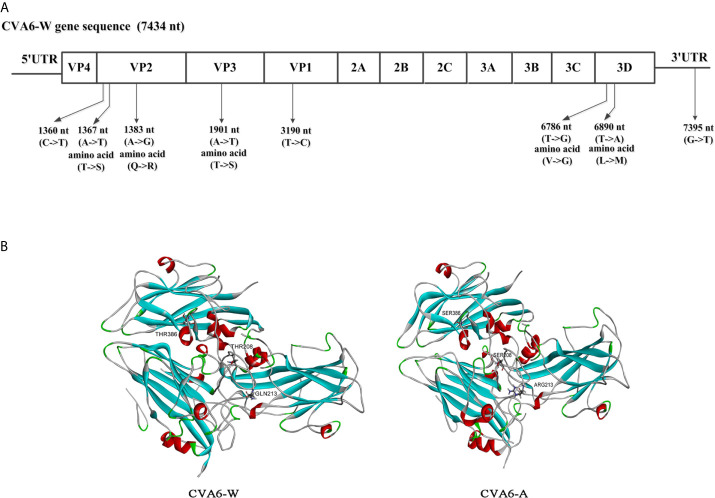
Comparative genomic analysis of the coxsackievirus A6 (CVA6) adapted strain (CVA6-A) with the parental CVA6 Gdula strain (CVA6-W). **(A)** Differences in nucleic acid and amino acid sequences are shown along with CVA6-W genomic locations and coding sequences. No insertion or deletion was found in CVA6-A genome. **(B)** Structural model of heterotrimeric capsid protein (VP2, VP3, and VP1) of CVA6-W and CVA6-A showing the variant amino acids.

To assess the genomic mutations of CVA6-A for adaption in mice, CVA6-A genome were sequenced, and each point mutation, and synonymous and nonsynonymous substitutions were identified and then compared by referencing CVA6-W. Compared with CVA6-W, strain CVA6-A had eight mutations (i.e., a G-to-T mutation in 3ˊ-UTR), two synonymous substitutions located in the coding regions of VP2 or VP1, three non-synonymous substitutions in the structural protein VP2 or VP3, and two substitutions in the non-structural protein 3D (**Figure 8A**). The CVA6-A and CVA6-W capsid models were built based on the template PDB 5YHQ of the CVA6 virus-like particle (VLP) Cryo-EM structure (**Figure 8B**). The capsids were composed of VP2, VP3, and VP1. All three mutated amino acids in VP2 and VP3 of CVA6-W were located on the capsid surface.

## Discussion

A superior animal model is needed to show the low cost, easy acquisition, easy operation advantages, and ability of simulating the pathogenesis of humans. Newborn mice have been used as a model of various enterovirus infections to preclinically evaluate vaccines (23–28). With the increase in CVA6 infection rate over the past few years, CVA6 isolated clinically was used to build the corresponding infection models in newborn mice by intraperitoneal, intramuscular, and intradermic injection methods (17), as an attempt to develop vaccines and antiviral drugs for preventing and treating CVA6. Compared with other injection routes, the intraperitoneal injection route can deliver higher doses of drugs and is easy to administer. CVA6-W could infect the mice, and the mice achieved significantly lower viral loads in the tissues than those in the group infected with CVA6-A. Thus, the stated result could probably explain why the survival curve, clinical symptoms, and increased weight of the mice in CVA6-W-challenged group were significantly inconsistent with those of the CVA6-A group. CVA6-W may be eliminated by the immune systems in the 10-day-old mice. CVA6-A could not trigger RD cells to produce CPE, which was suggested from the results obtained by using CVA6-A and CVA6-W with the identical virus content, to infect RD cells simultaneously. Accordingly, this study speculated that the mutated viral gene could cause mice to be vulnerable to the infection, and immunity against RD cells after CVA6-W was gradually adapted in newborn mice of different ages.

Moreover, the cytokines in the mice affected the infection efficiency of the virus, which might explain why the clinical isolates of CVA6-W and CVA6 or other enteroviruses could infect younger newborn mice. The IFN-γ concentration in the 3-day-old mice was higher than that of the mice of other ages, which is similar to the findings of existing studies on CVA4 infection model of newborn mice (19). It was verified that IFN-γ is capable of inducing cells to producing hWARS receptors, so the efficiency of enterovirus infection increases (29). A CVA6-infected mouse model exhibiting stable infection characteristics should be developed to effectively assess the immune effect of the CVA6 vaccine and the therapeutic effect exerted by antiviral drugs the more. Nevertheless, the newborn mice in most enterovirus infection models were younger, and this can impede CVA6 drugs or vaccine development because of the low tolerance of younger newborn mice to drugs and their inability to achieve active immunity.

A model of older newborn mice infected with CVA6 was built here and employed to assess the protective effect of recombinant IFN-α1b against CVA6. The advantages are presented below.

Advantage 1: The model is simple to operate, and the older mice are tolerable to a larger dose of the drug, the lethality rate of which was 100% for 10-day-old mice; so it can be further optimized.

Advantage 2: Viral genes could be detected in nervous tissues (e.g., cerebellums) of 10-day-old mice infected with the CVA6 mouse adapted strain, which indicates that the adapted strain may infect the nervous systems. The manifestations of children infected with enterovirus largely include neurological symptoms (e.g., encephalitis, encephalomyelitis, headache, vomiting, and limb shaking) (30). The previously built newborn mouse model is primarily characterized by phagocytosing muscles, and the clinical manifestation refers to hind limb paralysis. Whether the model built can be employed to study pathogenic mechanisms should be discussed in depth. With high similarity to the humans’ genetic maps, the genetic maps of non-human primates have been extensively used to study enteroviruses (31, 32), although the high cost and operational inconvenience of non-human primates limit their application. The built infection model of older newborn mice is economical to produce and easy to use to evaluate antiviral drugs subsequently.

Moreover, the animal model built here can be used to evaluate active immunization from vaccines. In most of the existing studies, adult mice have been used for the immunization with vaccines, and the serum adoptive method was adopted to evaluate the immune effect of enterovirus vaccines (33). Thus, the real effect of active immunity cannot be reflected, especially the evaluation of cellular immune effect (34, 35). Currently, children immunity is largely dependent on being inoculated with the EV71 inactivated vaccine (36). In this study, the 3-day-old mice were first immunized against the CVA6 inactivated virus, and then a challenge experiment was performed after 7 days. It was suggested that pre-immunization blocks EV71 infection, which is due to the previous effect of IgM produced by EV71 vaccine-induced mice (37). As indicated from recent clinical trials of the EV71 vaccine, it can be used for emergency vaccination for anti-EV71 infection (38). At present, the CVA6 vaccine has not been used clinically, so it is necessary to clarify the type of antibodies induced by the CVA6 vaccine and the level of neutralizing antibodies and its changes, for the early prevention of CVA6 infection. IgM was produced rapidly over time, and the antibody level tended to decrease after 7 days. Importantly, CVA6-specific IgG could be detected on day 7 post-immunization, and the IgG level increased on day 14 compared to that on day 7 post-immunization. In addition, CVA6 inactivated viruses were found to induce the production of protective neutralizing antibodies in newborn mice at an early stage after the immunization.

As demonstrated in previous studies in the past few years, certain key amino acid positions on the capsid protein of EV71 determine its replication ability in mice, as well as the susceptibility of mice of different ages to EV71, as well as the virulence of the viruses (39). As revealed in in-depth studies, certain vital amino acids on the EV71 VP1 protein show a relationship with the mild and severe clinical manifestations of diseases (40), and the mutations of some amino acid positions on the VP1 may cause neurological symptoms and severe HFMD (41). The number of cases of severe HFMD attributed to CVA6 have been rising over the past few years (42), and the mutations of certain amino acid positions on the CVA6 VP1 protein were suggested to correlate with the severity of HFMD (43).

In this study, CVA6-A was generated by infecting 10-day-old mice with CVA6 repeatedly. By performing the inoculation experiment on the mice, CVA6-A was obviously characterized by phagocytosing muscles, and the mice had clinical symptoms (e.g., hind limb paralysis) after 4 days. However, CVA6-W could not cause disease in 10-day-old mice. Here we found that there were eight mutations in the genome of CVA6-A compared with its parental CVA6 Gdula strain. It was speculated that certain single or multiple mutations caused significant differences in the virulence of CVA6 to mice. Previous studies have shown the protein kremen1 as the cell receptor for CVA6 and CVA10 (44). The crystal structures of CVA10 and human kremen1 protein complex indicate the binding of CVA10 VP1, VP2, and VP3 with human kremen1 protein (45). All three mutated amino acids in VP2 and VP3 of CVA6-A were located on the capsid surface (**Figure 8B**). The CVA6-A could not cause the RD cells CPE but could infect the 10-day-old mice. This result indicates one or more of the three mutated amino acids in VP2 and VP3 may be critical for CVA6-W binding with human kremen1 protein. One or more of the three amino acids mutated in VP2 and VP3 may enhance the CVA6-A binding with mouse kremen1 protein, which would be investigated in our future work. Other mutations may also be associated with the increased virulence of CVA6-A in mouse, which could be further studied in detail. In our future works, these would be researched with a panel of mutant viruses.

CVA6 strains are segregated into four distinct genotypes designated as A, B, C, and D based on the phylogenetic tree of VP1 gene (46, 47). D3 is the predominant sub-genotype that circulates in recent years in mainland China (48). In this study the CVA6 reference strain, Gdula strain (genotype A), was used to obtain CVA6-A strain. We found that capsid proteins in different CVA6 genotypes are conserved by amino acid sequence alignment. The percent identity of amino acid sequences of capsid proteins (VP1-4) is about 95.6% between D3 and A strains. The VP1 protein of CVA6 is the main neutralizing antigen, and Xu et al. mapped an immune-dominant neutralizing epitope to the surface loops of VP1 of CVA6 (49). There are two amino acid differences in the epitope region between D3 and A strains. However, there is no report of cross-neutralization between different CVA6 genotypes. The 10-day-old murine model built here on CVA6-A strain may be used to evaluate the cross-neutralization to different CVA6 genotypes, especially to the epidemic CVA6 strains.

The CVA6-A virus can still only infect the suckling mice but not the adult mice. However, CVA6 is clinically pathogenic in children but not in adults, which may be partially explained by the immature immune status of children. Therefore, suckling mouse may be better to mimic children infected by CVA6. Caine et al. obtained mouse-adapted strains of CVA6 through mice deficient in IFN-α/β receptors (A129), which could infect the 3-week-old A129 mice. Protection from CVA6 challenge was accomplished through a passive transfer study (50). Here the normal mouse-adapted strain, CVA6-A, may infect older immune deficient mice such as A129 or AG129 for active immunization studies, which could be investigated in future.

In brief, a CVA6 adapted strain capable of sacrificing 10-day-old mice and causing typical clinical symptoms in newborn mice was generated by infecting the newborn mice of different ages repeatedly. Lastly, a model of 10-day-old mice infected with CVA6 was built. As indicated by the IFN-α1b treatment experiment, compared with newborn mouse infection models built by others, the animal model built in this study could receive higher doses of drug treatments. In addition, as revealed by the monitoring of the early immune response induced by the CVA6 inactivated virus and by adopting the built infection model of the newborn mice, the CVA6 inactivated virus could be used as emergency vaccination. The present study can help build an evaluation model to develop anti-CVA6 drugs and vaccines.

## Data Availability Statement

The original contributions presented in the study are included in the article/supplementary material. Further inquiries can be directed to the corresponding authors.

## Ethics Statement

The animal study was reviewed and approved by the laboratory animal ethics committee of Guangdong Medical University.

## Author Contributions

Conception and design: ZJ and XT. Investigation: ZJ, YZ, HL, QC, WL, and XL. Interpretation of data: ZJ and XT. Writing-original draft: ZJ and XT. Writing-review and editing: ZJ, YZ, HL, QC, WL, XL, XT, and BZ. Supervision and project administration: XT and BZ. Funding acquisition: XT and BZ. All authors contributed to the article and approved the submitted version.

## Funding

Funding support was provided by the National Key Research and Development Program of China (2018YFC1200100) to XT, the National Natural Science Foundation of China (82072264) to XT, the Natural Science Foundation of Guangdong Province (2019A1515011514) to BZ, Guangdong Medical Science Research Foundation (B2020187) to BZ, the Independent Project of the State Key Laboratory of Respiratory Disease (SKLRD-Z-202113) to ZJ, and the Post-doctoral Foundation of First Affiliated Hospital of Guangzhou Medical University to ZJ.

## Conflict of Interest

The authors declare that the research was conducted in the absence of any commercial or financial relationships that could be construed as a potential conflict of interest.
